# Quality assurance of human papillomavirus (HPV) testing in the implementation of HPV primary screening in Norway: an inter-laboratory reproducibility study

**DOI:** 10.1186/s12879-016-2028-7

**Published:** 2016-11-24

**Authors:** Birgit Engesæter, Bianca van Diermen Hidle, Mona Hansen, Pia Moltu, Kjersti Mangseth Staby, Siri Borchgrevink-Persen, Olav K. Vintermyr, Stefan Lönnberg, Mari Nygård, Emiel A. M. Janssen, Philip E. Castle, Irene Kraus Christiansen

**Affiliations:** 1 Cancer Registry of Norway, Oslo, Norway; 2 Department of Pathology, Stavanger University Hospital, Stavanger, Norway; 3 Department of Microbiology and Infection Control, Akershus University Hospital, Lørenskog, Norway; 4 Department of Pathology, Haukeland University Hospital, Bergen, Norway; 5 Department of Pathology and Medical Genetics, St. Olavs Hospital, Trondheim University Hospital, Trondheim, Norway; 6 The Gade Laboratory for Pathology, Department of Clinical Medicine (K1), University of Bergen, Bergen, Norway; 7 Department of Mathematics and Natural Sciences, University of Stavanger, Stavanger, Norway; 8 Albert Einstein College of Medicine, Bronx, NY USA

**Keywords:** Human papillomavirus, HPV, Cervical cancer screening, HPV primary screening, HPV testing, Quality assurance, Cobas 4800 HPV Test, Inter-laboratory reproducibility

## Abstract

**Background:**

Human papillomavirus (HPV) testing as primary screening for cervical cancer is currently being implemented in Norway in a randomized controlled fashion, involving three laboratories. As part of the quality assurance programme of the implementation, an evaluation of the inter-laboratory reproducibility of the HPV test was initiated, to ensure satisfactory HPV test reliability in all three laboratories.

**Methods:**

The HPV test used is the cobas 4800 HPV Test, detecting 14 high-risk types with individual HPV genotype results for HPV16 and HPV18. In addition to the three laboratories involved in the implementation, the Norwegian HPV reference laboratory was included as a fourth comparative laboratory. A stratified sample of 500 cervical liquid based cytology (LBC) samples was used in the evaluation, with an aim towards a high-risk HPV positivity of ~25%. Samples were collected at one laboratory, anonymized, aliquoted, and distributed to the other laboratories.

**Results:**

Comparison of the test results of all four laboratories revealed a 95.6% agreement, an 86.3% positive agreement and a kappa value of 0.94 (95% CI 0.92–0.97). For negative cytology specimens, there was a 95.8% overall agreement, a 67.4% positive agreement, and a kappa value of 0.88 (95% CI 0.80–0.93). For abnormal cytology specimens, there was a 95.8% overall agreement, a 95.5% positive agreement, and a kappa value of 0.86 (95% CI 0.71–0.97).

**Conclusions:**

The study showed a high inter-laboratory reproducibility of HPV testing, implying satisfactory user performance and reliability in the laboratories involved in the implementation project. This is important knowledge and we recommend similar studies always to be performed prior to the introduction of new screening routines.

**Electronic supplementary material:**

The online version of this article (doi:10.1186/s12879-016-2028-7) contains supplementary material, which is available to authorized users.

## Background

Identification of the association between high-risk human papillomavirus (HR-HPV) and cervical carcinogenesis has led to revolutionary advances in cervical cancer screening, including sensitive, molecular HPV tests that improve early detection of cervical precancerous lesions. In Norway, HPV testing was introduced in triage of women diagnosed with ASC-US (atypical squamous cells of undetermined significance) and LSIL (low-grade squamous intraepithelial lesions) in 2005. Based on randomized clinical trials of HR-HPV detection conducted in Europe, as well as recommendations by the European guidelines for quality assurance in cervical cancer screening [1–3], HPV testing in primary screening has been introduced in several European countries during the last few years. The more sensitive and objective HPV test is proposed to increase health benefit and reduce the harms associated with screening. In addition, the objectivity of the test implies better reproducibility compared to cytology [4]. Still, primary HPV-based screening has been and is controversial, related to the low clinical specificity of HPV DNA tests. HPV DNA testing will identify several infections never leading to dysplasia or cancer, especially in younger age-groups. Of this reason, HPV primary screening is commonly introduced for women above the age of 30. Also, low specificity of HPV DNA testing is partly compensated for by cytological triage of HPV positive women (http://www.kreftregisteret.no/hpv-algoritme).

In 2015, gradual implementation of primary HPV screening in the Norwegian cervical cancer screening programme was initiated. Women in the age group 34–69 years resident in four (of a total of 19) counties in Norway, are randomized to HPV testing or cytology according to their date of birth. The HPV analyses are performed by three laboratories located at Stavanger University Hospital, Stavanger, Haukeland University Hospital, Bergen and St. Olavs Hospital, Trondheim University Hospital, Trondheim. After competitive bidding, the cobas ﻿4800 HPV Test (Roche Diagnostics) was selected for the implementation. In 2014, the cobas HPV Test was U.S. Food and Drug-approved for use in primary cervical cancer screening for women 25 year and older.

In addition to good clinical performance, reproducibility of laboratory results is fundamental for safe screening. To confirm satisfactory HPV test reproducibility and user performance within the screening programme, an evaluation of inter-laboratory reproducibility between the three service laboratories involved in the implementation was performed. The Norwegian HPV reference laboratory (Akershus University Hospital, Lørenskog), with cobas 4800 HPV Test accreditation since 2011, contributed as a fourth evaluator. Two of the three service laboratories had no previous experience with the test, and a shared laboratory handbook containing necessary standard operating procedures (SOPs) for HPV testing was prepared to ensure standardized methods. The inter-laboratory evaluation of HPV testing reported here was considered part of the quality assurance programme of the implementation of HPV primary screening in Norway, aiming to confirm satisfactory user performance and reliability in each of the three laboratories.

## Methods

### Clinical material and study design

Residual material of cervical liquid based cytology (LBC) samples (in PreservCyt medium, Hologic, Bedford, MA, USA) taken as part of the cervical cancer screening programme in Norway, was used. The samples were collected at Stavanger University Hospital, Department of Pathology, in the time period December 16th 2014 to May 21st 2015. All ages were included (comprising ages 16–81 years).

Two-sided Z test (kappa statistic) for agreement between two rates was used for sample size determination [5]. Power calculation showed that a sample size of 480 subjects achieved 86% power to detect a true Kappa value of 0.80 in a test of H0: Kappa = 0.69 vs. H1: Kappa <> 0.69. The power calculation was based on a significance level of 0.05. A total of 500 samples were collected with a composition aiming to provide an approximate prevalence of high-risk HPV of 25%. In order to reach that goal, 100 samples were selected based on the cytological results, either high-risk HPV DNA positive ASC-US or LSIL samples; or HSIL (high-grade squamous intraepithelial lesion) or ASC-H (atypical squamous cells, cannot rule out HSIL), assumed to be HPV positive. In addition, 400 consecutive samples without regard to their cytological results were included.

For ethical reasons, all samples were anonymized before HPV analysis. Cytological diagnoses were recorded prior to anonymization, important for the final evaluation of the results. For HSIL and ASC-H samples, a high concordance is essential. For NILM (negative for intra-epithelial lesions or malignancy) samples, with a generally lower viral load expected, a certain degree of discordance is expected due to the stochasticity of the PCR process. Due to anonymization of the samples, review of cytological diagnoses and/or comparison to histological results was not feasible.

One sample was excluded from the analyses due to empty sample tube upon arrival at one laboratory. Hence, the presented data are based on results from 499 samples.

Considering all 499 samples, 24% presented samples with cellular changes (ASC-US 7%, LSIL 7%, ASC-H 2% and HSIL 8%), while 5% was unsatisfactory for cytological evaluation. The sample distribution according to cytological result is given in Table 1.

**Table 1:** Distribution of cytological diagnoses among the 499 samples

	NILM *N* = 355	ASC-US *N* = 36	LSIL *N* = 33	ASC-H *N* = 11	HSIL *N* = 38	Unsatisfactory *N* = 27
100 selected samples	0	28^a^	28^a^	11	33	0
399 consecutive samples	354	8	5	0	5	27

^a^HPV positive samples

*NILM* negative for intraepithelial lesion or malignancy, *ASC-US* atypical squamous cells of undetermined significance, *LSIL* low-grade squamous intraepithelial lesion, *ASC-H* atypical squamous cells, cannot exclude a high-grade lesion, *HSIL* high-grade squamous intraepithelial lesion

### HPV determination

The cobas 4800 HPV Test is a fully automated, qualitative multiplex assay, including full sample preparation (cobas x 480) and HPV detection (cobas z 480 Analyser) based on real-time polymerase chain reaction (PCR) technology. The method detects 14 high-risk types, including individual HPV genotype results for HPV16 and HPV18. The other 12 types are reported concurrently by a pooled result (HR-HPV). Beta-globin is used as an internal quality control for each sample. The system is provided with an advanced result algorithm for the calculation of distinct HPV positive, HPV negative or invalid (negative beta-globin) results and the ability to review reaction curves manually is not routinely available for users. In addition, of various indicated reasons, the sample may come up as “failed”, with the most common explanations being clots or insufficient sample volume.

Results are reported through four channels specific for HPV16, HPV18, HR-HPV, or beta-globin, respectively. Each channel has a distinct cut-off value, based on cycle threshold (Ct) values to distinguish a clinically positive from a negative sample. The cut-off values are 40.5, 40.0, 40.0 and 40.0 for the four channels HPV16, HPV18, HR-HPV and beta-globlin, respectively [6]. Sets of positive controls (one for each channel; HPV16, HPV18 and HPV39) and negative controls are included for each set-up. The 960 sample kit was used by all laboratories.

### Preparation and distribution of clinical material

Cytology was interpreted according to the Bethesda system as unsatisfactory, NILM, ASC-US, LSIL, ASC-H and HSIL. After registration of cytological diagnosis, the samples were anonymized and given a study identification number. The samples were stored within the validated storage parameters ascribed by the manufacturer, which says a maximum of 6 months at 2–30 °C. Each LBC sample was vigorously mixed by using a vortex machine and aliquoted to four Sarstedt tubes (1.25 ml to each tube allowing two runs with the cobas HPV Test). The first set of aliquots was analysed by lab A, the second by lab B, the third by lab C and the fourth by lab D (The Norwegian HPV reference laboratory).

### Performance at the individual laboratory

Each laboratory (A, B, C and D) tested one set of aliquots blinded for all information except for the study identification number. The samples were run within the same defined period of two weeks. Samples were scanned directly onto the instrument and a work-list (“work order”) was created. The analysis was performed according to the shared SOPs (www.kreftregisteret.no/laboratoriemappe). Samples obtaining “failed” results in the first analysis were rerun once. If persistent, the sample was reported as failed, and a flag explaining the reason for not being analyzed was attached. Remaining sample material after analysis was discarded. Results (raw data) of the testing from each lab were exported to Excel (Microsoft Office) and sent to the Cancer Registry of Norway for analysis and interpretation.

### Statistical analyses

The inter-laboratory reproducibility was assessed by comparison of the test results using percentage agreement, positive percentage agreement and Fleiss’ kappa statistics. Differences in test results were compared pairwise between the laboratories using McNemar test. Stata version 14.1 (Stata Corporation, College Station) was used for the analyses.

## Results

HPV test results showed a mean HPV positivity rate of 28.9% for the four laboratories, varying from 28.3 to 29.3% (Table 2). Table 2 shows the frequency of HPV negative, HPV positive and failed samples for the laboratories, stratified on the cytological categories NILM, ASC-US, LSIL, ASC-H/HSIL and unsatisfactory.

**Table 2:** Frequencies of HPV negative, HPV positive and failed samples considering all 499 samples, stratified on cytological categories

	All (*n* = 499)			NILM (*n* = 354)			ASC-US (*n* = 36)			LSIL (*n* = 33)			ASC-H/HSIL (*n* = 49)			Unsatisfactory (*n* = 27)			ASC-US or more severe (*n* = 118)		
	#pos (%)	#neg (%)	#failed (%)	#pos (%)	#neg (%)	#failed (%)	#pos (%)	#neg (%)	#failed (%)	#pos (%)	#neg (%)	#failed (%)	#pos (%)	#neg (%)	#failed (%)	#pos (%)	#neg (%)	#failed (%)	#pos (%)	#neg (%)	#failed (%)
Lab A	145 (29.1)	350 (70.1)	4 (0.8)	34 (9.6)	317 (89.6)	3 (0.8)	32 (88.9)	4 (11.1)	0 (0.0)	33 (100.0)	0 (0.0)	0 (0.0)	44 (89.8)	5 (10.2)	0 (0.0)	2 (7.4)	24 (88.9)	1 (3.7)	109 (92.4)	9 (7.6)	0 (0.0)
Lab B	145 (29.1)	346 (69.3)	8 (1.6)	34 (9.6)	314 (88.7)	6 (1.7)	33 (91.7)	3 (8.3)	0 (0.0)	31 (93.9)	0 (0.0)	2 (6.1)	44 (89.8)	5 (10.2)	0 (0.0)	2 (11.1)	24 (88.9)	0 (0.0)	108 (91.5)	8 (6.8)	2 (1.7)
Lab C	141 (28.3)	355 (71.1)	3 (0.6)	32 (9.0)	321 (90.7)	1 (0.3)	31 (86.1)	4 (11.1)	1 (2.8)	32 (96.8)	0 (0.0)	1 (3.0)	44 (89.8)	5 (10.2)	0 (0.0)	2 (7.4)	25 (92.6)	0 (0.0)	107 (90.7)	9 (7.6)	2 (1.7)
Lab D	146 (29.3)	353 (70.7)	0 (0.0)	35 (9.9)	319 (90.1)	0 (0.0)	32 (88.9)	4 (11.1)	0 (0.0)	33 (100.0)	0 (0.0)	0 (0.0)	44 (89.8)	5 (10.2)	0 (0.0)	2 (7.4)	25 (92.6)	0 (0.0)	109 (92.4)	9 (7.6)	0 (0.0)
% Overall Agreement	95.6 (93.8–97.2)			95.8 (93.5–97.7)			94.4 (86.1–100.0)			90.9 (78.8–100.0)			100.0 (100.0–100.0)			92.6 (81.5–100.0)			95.8 (92.4–99.2)		
% Positive Agreement	90.8 (85.9–94.9)			79.4 (65.1–91.5)			93.9 (84.8–100.0)			90.9 (78.8–100.0)			100.0 (100.0–100.0)			66.7 (0.0–100.0)			95.5 (91.4–99.1)		
Kappa	0.94 (0.92–0.97)			0.88 (0.80–0.93)			0.86 (0.52–1.00)			n/a			1.0			0.78 (−0.03–1.00)			0.86 (0.71–0.97)		

Agreement statistics with 95% confidence intervals between the laboratories are shown

*NILM* negative for intraepithelial lesion or malignancy, *ASC-US* atypical squamous cells of undetermined significance, *LSIL* low-grade squamous intraepithelial lesion, *ASC-H* atypical squamous cells, cannot exclude a high-grade lesion, *HSIL* high-grade squamous intraepithelial lesion

Agreement between the laboratories was calculated by overall percentage agreement, positive percentage agreement and Fleiss’ kappa. Comparison of the results from all four laboratories revealed 95.6% overall agreement, 86.3% positive agreement and a kappa value of 0.94 (95% CI 0.92–0.97) (Table 2). A pairwise comparison (not adjusted for multiple comparisons) did not reveal a significant difference between the results from the four laboratories (*p* > 0.05). Reproducibility between the laboratories was not dependent on cytological interpretation. Notably, a 100% agreement was obtained for the samples with the most severe cytological interpretation (ASC-H and HSIL) (Table 2). The lowest inter-laboratory agreement was observed among results from women with unsatisfactory cytology (*n* = 27) with the overall percent agreement of 92.6 and a kappa value of 0.78.

Of the 499 samples, 22 samples showed discordant results (Table 3). For 19 samples, one of the four laboratories showed discordancy compared to the three other laboratories. For two samples, two and two laboratories had the same results. Only one sample showed three different results (HPV positive, HPV negative and failed). Two categories of discordant results were observed; 1) Discordancy in terms of a failed sample for at least one laboratory (*n* = 12), and 2) Discordancy in terms of HPV positive vs HPV negative results (*n* = 10). Comparison of the test results from all four laboratories, excluding the failed samples, showed 98.2% overall agreement, 93.9% positive agreement and a kappa value of 0.98 (95% CI 0.96–0.99). The four involved laboratories reported in total 15 failed samples. In a clinical setting, these women would be recalled to take a new cell sample from the cervix. Almost all of these samples (14/15) came up with the flag X3 “Clot was detected; Sample was not processed”.

**Table 3:** Samples with discordant HPV test results (HPV positive, HPV negative, failed) between the four laboratories

Cytological finding	Lab A	Lab B	Lab C	Lab D	
Unsatisfactory	failed	negative	negative	negative	*
NILM	failed	negative	negative	negative	*
NILM	failed	negative	failed	negative	*
NILM	negative	failed	negative	negative	*
NILM	negative	failed	negative	negative	*
NILM	negative	failed	negative	negative	*
NILM	negative	failed	negative	negative	*
NILM	negative	failed	negative	negative	*
ASCUS	positive	positive	failed	positive	*
LSIL	positive	failed	positive	positive	*
LSIL	positive	failed	positive	positive	*
LSIL	positive	positive	failed	positive	*
Unsatisfactory	negative	positive	negative	negative	**
NILM	failed	failed	negative	positive	**
NILM	positive	negative	negative	negative	**
NILM	positive	positive	negative	negative	**
NILM	positive	positive	positive	negative	**
NILM	negative	positive	negative	negative	**
NILM	negative	positive	negative	positive	**
NILM	negative	negative	negative	positive	**
NILM	negative	negative	negative	positive	**
ASCUS	negative	positive	negative	negative	**

*Samples for which at least one laboratory reported a failed result, and the women would be recalled to take a new call sample from the cervix

** True discordant samples; would lead to different clinical follow-up procedures

*NILM* negative for intraepithelial lesion or malignancy, *ASC-US* atypical squamous cells of undetermined significance, *LSIL* low-grade squamous intraepithelial lesion

For the 10 true discordant samples, different follow-up procedures would be recommended; seven of the samples had NILM cytology, two ASC-US, three LSIL and one unsatisfactory, emphasizing that none had severe cytological abnormalities (ASC-H or HSIL). The Ct-values of the discordant samples (Additional file 1: Table S1) were generally above 38.4 (with two exceptions), and hence close to the clinical Ct cut-off values. The Ct-values of beta-globin were for all samples between 25.5 and 28, indicating sufficient quality of the input DNA.

The cobas HPV Test detects HPV16, HPV18 and 12 additional high-risk types in a pooled analysis. In total, nine samples showed discordant genotype results (Additional file 2: Table S2). No samples showed complete discordance, i.e., there was always partial concordance between the genotypes. Concordance after including genotype information, showed 93.8% overall agreement, 78.7% positive agreement and a kappa value of 0.93 (95% CI 0.91–0.96) (Table 3). Calculation was also performed stratifying multiple infections according to oncogenic potential of the HPV types present; HPV16>HPV18>HR-HPV. The results showed 94.4% overall agreement, 82.5% positive agreement and a kappa value of 0.94 (95% CI 0.91–0.96) (Table 4).

**Table 4:** Agreement of HPV test results between the four laboratories including HPV genotype information

Test results compared	Overall percentage agreement	Positive percentage agreement	*k* value (95% CI)	Number of samples
Multiple genotypes taken into consideration	93.8	78.7	0.93 (0.91–0.96)	499
Highest risk genotype taken into consideration	94.4	82.5	0.94 (0.91–0.96)	499

Calculations were performed both with and without taking multiple infections into consideration. For calculation not considering multiple infections, HPV type category with highest oncogenic potential was the deciding factor; HPV16>HPV18>HR-HPV

## Discussion

Safe implementation of primary HPV testing in cervical screening programmes is dependent on reliable test results to maximize the benefits of HPV testing. Currently, implementation of primary HPV screening is on-going in Norway, involving women resident in four counties (and three laboratories). The screening interval for women with a negative HPV test is 5 years, compared to 3 years with cytology, and endeavors to minimize false negative results is particularly important. An evaluation of the inter-laboratory reproducibility of the HPV testing was initiated as part of the quality assurance programme. In addition to the three laboratories involved in the implementation, the Norwegian HPV reference laboratory contributed as a fourth comparing laboratory. For yearly quality control, the laboratories are attending the Quality Control for Molecular Diagnostics (QCMD) HPV DNA external quality assessment (EQA) programme.

In general, the HPV testing showed high reproducibility between the four sites. Both overall, and after stratification according to cytological diagnoses and HPV genotypes, high agreement between the laboratories was observed, with kappa values above 0.85. The overall agreement including all samples was 95.6% (95% CI 93.8–97.4%) and the kappa value was 0.95. These results are well above the recommended lower confidence bound of 87% overall agreement and kappa value of at least 0.5, suggested by “Guidelines for HPV test DNA requirement for primary cervical cancer screening” authored by Meijer et al. [7].

Detection of HPV is dependent on the number of viral DNA copies present in the sample. A negative test result does not guarantee absence of HPV DNA in the sample, just that the level is below a defined threshold value based on the clinical characteristics of the test [8]. In this evaluation, ten samples showed positive vs negative discordant results, which according to the current screening algorithm would result in different follow-up of the women (http://www.kreftregisteret.no/hpv-algoritme). For the discordant results, samples with HPV positive outcomes had high Ct-values (>38.4) with two exceptions, implying low levels of viral DNA. The discrepancy in test results (HPV negative vs HPV positive) may be associated with the inherent stochasticity of the PCR process, i.e., whether the Ct-value will be below or above the clinical Ct-threshold value for samples with low viral load, may be related to coincidence. Accordingly, discordant results may also be explained by intra-laboratory variability.

All samples with a severe cytological diagnosis (ASC-H or HSIL) had concordant results. This is regarded as a significant finding due to the importance in identifying these women when HPV testing is introduced as a primary screening test. High-grade lesions generally have higher viral loads, possibly also explaining the higher concordance rate among these samples. It is noteworthy however, that four HSIL samples had an HPV negative test result in all four laboratories. Based on the close relation between HSIL and HPV infection, false positive cytology results or false negative HPV results are likely explanations. HPV testing, although sensitive, may not capture all cases of disease and hence, these may be true false negative cases. Due to the anonymisation of samples, recalling of the cytology results and/or comparison with histological data was, however, not feasible. In the end, given the aim of this study, the concordance in test results between the laboratories was considered a central finding.

According to the algorithm for primary HPV screening in Norway, HPV genotypes are not reported and all HPV-positive women have a cytological reflex evaluation. Still, categorizing the genotypes based on the cobas HPV Test results, may be of interest as it has been suggested that HPV16 and/or HPV18 positive women should have a more aggressive follow-up regime compared to women positive for the HR-HPV category [9]. In the present data, discordant results related to genotypes were observed for nine samples. Discordance in terms of HPV16/18 versus HR-HPV, is seen for four samples. Discrepancy in genotype results may be related to the multiplex PCR process where targets in the reaction compete with each other for resources.

The cobas 4800 instrument automatically “approves” the results based on algorithmic criteria in the software. Accordingly, samples may fail to provide a result. Failed samples are often explained by insufficient material, too much material or cellular aggregates, or may be a result of insufficient mixing of the samples before loading on the instrument. Failed samples were rerun, and upon a second fail reported as failed in the study report. This is a common procedure also in routine screening, where the women are recalled for a new sample after two failed analyses. For 11 of the 13 samples reported as “failed” (Table 3), the failure was only reported from one laboratory, suggesting the failure being related to technical issues in the laboratory rather than to the quality of the sample. Samples should before loading always be visually inspected, evaluating the need for additional mixing or even removal of cellular aggregates. For primary screening, this is however not always feasible due to high quantity of samples and use of the instrument cobas p 480 for automated sample handling. A suggested procedure would be to evaluate samples prior to reloading after a failed result.

Along with the implementation of primary HPV testing, proper directions for quality assessment is paramount. In Norway, several initiatives are on-going. Centralization of HPV testing and cytology reading is in process to ensure a sound environment with high molecular biology competence for HPV testing and with sufficient amount of cytology samples to satisfy “the European guidelines for quality assurance in cervical cancer screening” of 15,000 samples per year for each analyzing laboratory [3]. Laboratories performing HPV testing should have, and maintain, accreditation to ISO15189 standard. Among others, this involves that each laboratory should have SOPs describing all steps from sample arrival to report. In addition, internal quality assurance (ICA) should ensure that systematic method verification is performed and approved upon the implementation of a new HPV test, documenting for example internal reproducibility of the assay. Also, the introduction of kit independent controls should be considered, which is important not only as an independent control for each run, but also for the verification of analytical stability over time. As a necessary supplement to ICA, all laboratories should regularly participate to international EQA programmes in order to evaluate and document adequate performance, as the QCMD HPV DNA programme. There is, however, a prominent need for additional panels specifically designed for HPV-based screening, evaluating performance based on clinical cut-off values in the test. Importantly, such a proficiency screening panel is planned by the WHO HPV LabNet-programme [10], which would constitute an important contribution to quality assurance of HPV-based cervical cancer screening. Awaiting such a panel, Norway will, in addition to participation in the QCMD programme, continue with circulation of samples between laboratories every second year as part of the continuous quality assurance of the screening programme. Essentially, we support the message given by Carozzi and collegues [11], emphasizing the need for international efforts in order to establish quality guidelines in line with the change to HPV-based screening in several countries.

### Study constraints

For ethical reasons, all samples were anonymized before HPV-analysis and consequently, comparison to histological results was not feasible. This would have been valuable in order to better explain the HPV negative HSIL samples observed. In the implementation pilot, only the cobas HPV Test is used and cell samples are collected in PreservCyt media. Lacking assessment of reproducibility between several HPV tests and transport media entails a limitation to the generality of this evaluation, but fulfil the requirement for the Norwegian implementation.

## Conclusions

Transition from HPV testing as a secondary screening test to primary HPV testing requires a significant reorganization of laboratory activities. Laboratory staff needs good information and training to be able to contribute at a high professional level. The assessment of the inter-laboratory reproducibility of the HPV testing is an important contribution to the quality assurance. Overall, the results in this study show a high inter-laboratory reproducibility of the HPV testing with the cobas HPV Test, certifying satisfactory laboratory performance in each of the three laboratories involved in the implementation of HPV primary screening in Norway. This is important knowledge and we recommend similar studies always to be performed prior to the introduction of new screening routines.

## Additional files

Additional file 1: Table S1. Ct-values for HPV and beta-globin detection in samples with discordant results. The Ct-values of the discordant samples were generally above 38.4 (with two exceptions), and hence close to the clinical Ct cut-off values. The Ct-values of beta-globin were for all samples between 25.5 and 28, indicating sufficient quality of the input DNA. (DOCX 18 kb)
Additional file 2: Table S2. Discordant genotype results among HPV positive samples. Nine samples showed discordant genotype results. No samples showed complete discordance, i.e., there was always partial concordance between the genotypes. (DOCX 15 kb)

## Abbreviations

ASC-H: Atypical squamous cells, cannot rule out HSIL
ASC-US: Atypical squamous cells of undetermined significance
EQA: External quality assessment
HPV: Human papillomavirus
HR-HPV: High-risk human papillomavirus
HSIL: High-grade squamous intraepithelial lesions
IQA: Internal quality assurance
LBC: Liquid based cytology
LSIL: Low-grade squamous intraepithelial lesions
NILM: Negative for intra-epithelial lesions or malignancy
QCMD: Quality control for molecular diagnostics
SOP: Standard operating procedure
