# Supplementary material for: Quality assurance of human papillomavirus (HPV) testing in the implementation of HPV primary screening in Norway: an inter-laboratory reproducibility study
Source: BMC Infect Dis. 2016 Nov 24;16:698. doi: 10.1186/s12879-016-2028-7 (PMC5122146; doi:10.1186/s12879-016-2028-7)
Supplement: Additional file 1: Table S1. — Ct-values for HPV and beta-globin detection in samples with discordant results. The Ct-values of the discordant samples were generally above 38.4 (with two exceptions), and hence close to the clinical Ct cut-off values. The Ct-values of beta-globin were for all samples between 25.5 and 28, indicating sufficient quality of the input DNA. (DOCX 18 kb) [file 12879_2016_2028_MOESM1_ESM.docx]

**Table S1.** Ct-values for HPV and beta-globin detection in samples with discordant results

| **Sample ID** | **Lab A** | | **Lab B** | | **Lab C** | | **Lab D** | |
| --- | --- | --- | --- | --- | --- | --- | --- | --- |
|  | HPV | Betaglobin | HPV | Betaglobin | HPV | Betaglobin | HPV | Betaglobin |
| 5 | 38.8^2^ | 25.5" | 39.4^2^ | 28.0 | 38.9^2^ | 26.2 | - | 27.8 |
| 11 | 40.0^3^ | 26.0" | - | 26.5 | - | 26.7 | - | 26.6 |
| 83 | - | 25.3" | 40.0^3^ | 25.7 | - | 25.5 | - | 26.6 |
| 176 | - | 27.8" | - | 27.6 | - | 26.8 | 39.5^2^ | 26.8 |
| 231 | 38.4^3^ | 25.8" | 35.7^3^ | 25.8 | - | 26.0 | - | 25.9 |
| 270 | - | 26.9" | 37.7^3^ | 26.1 | - | 26.7 | - | 26.7 |
| 361 | - | 25.9" | - | 24.9 | - | 25.4 | 40.2^1^ | 25.7 |
| 435 | - | 27.7" | 39.5^3^ | 27.3 | - | 27.7 | - | 27.4 |
| 450 | - | 28.3" | - | 27.8 | - | 28.1 | 39.5^3^ | 27.0 |
| 484 | N/A | N/A | N/A | N/A | - | 26.4 | 39.9^3^ | 26.6 |

*^1)^ Ct value for HPV16 detection*

*^2)^ Ct value for HPV18 detection*

*^3)^ Ct value for HR-HPV detection*

*Clinical cut-off values for HPV16, HPV18 and HR-HPV are 40.5, 40.0 and 40.0, respectively [6]*
