# Supplementary material for: Quality assurance of human papillomavirus (HPV) testing in the implementation of HPV primary screening in Norway: an inter-laboratory reproducibility study
Source: BMC Infect Dis. 2016 Nov 24;16:698. doi: 10.1186/s12879-016-2028-7 (PMC5122146; doi:10.1186/s12879-016-2028-7)
Supplement: Additional file 2: Table S2. — Discordant genotype results among HPV positive samples. Nine samples showed discordant genotype results. No samples showed complete discordance, i.e., there was always partial concordance between the genotypes. (DOCX 15 kb) [file 12879_2016_2028_MOESM2_ESM.docx]

**Table S2.** Discordant genotype results among HPV positive samples

| **Sample ID** | **Cytological diagnosis** | **Lab A** | **Lab B** | **Lab C** | **Lab D** |
| --- | --- | --- | --- | --- | --- |
| 92 | ASC-US | HR-HPV | HR-HPV | HR-HPV | HPV16 & HR-HPV |
| 147 | ASC-US | HR-HPV | HR-HPV | HPV16 & HR-HPV | HR-HPV |
| 212 | LSIL | HR-HPV | HR-HPV | HPV16 & HR-HPV | HR-HPV |
| 372 | ASC-US | HPV16 & HR-HPV | HPV16 & HR-HPV | HPV16 & HR-HPV | HR-HPV |
| 452 | ASC-H | HPV18 & HR-HPV | HPV18 & HR-HPV | HPV18 | HPV18 |
| 462 | ASC-H | HPV16 | HPV16 | HPV16 | HPV16 & HR-HPV |
| 477 | HSIL | HPV16, HPV18 & HR-HPV | HPV18 & HR-HPV | HPV16, HPV18 & HR-HPV | HPV18 & HR-HPV |
| 482 | ASC-H | HPV18 | HPV16 & HPV18 | HPV16 & HPV18 | HPV18 |
| 496 | NILM | HPV16 & HR-HPV | HPV16 & HR-HPV | HPV16 | HPV16 |

*NILM – negative for intraepithelial lesion or malignancy*

*ASC-US – atypical squamous cells of undetermined significance*

*LSIL – low-grade squamous intraepithelial lesion*

*ASC-H – atypical squamous cells, cannot exclude a high-grade lesion*

*HSIL – high-grade squamous intraepithelial lesion*
